# Mechanical Properties of Epoxy Resin Mortar with Sand Washing Waste as Filler

**DOI:** 10.3390/ma10030246

**Published:** 2017-02-28

**Authors:** Dinberu Molla Yemam, Baek-Joong Kim, Ji-Yeon Moon, Chongku Yi

**Affiliations:** School of Civil, Environmental and Architectural Engineering, Korea University, Anam-dong, Seongbuk-gu, Seoul 02841, Korea; yaatdg@gmail.com (D.M.Y.); kaka790905@korea.ac.kr (B.-J.K.); yeon414@korea.ac.kr (J.-Y.M.)

**Keywords:** epoxy resin mortar, polymer binder, filler, mechanical properties

## Abstract

The objective of this study was to investigate the potential use of sand washing waste as filler for epoxy resin mortar. The mechanical properties of four series of mortars containing epoxy binder at 10, 15, 20, and 25 wt. % mixed with sand blended with sand washing waste filler in the range of 0–20 wt. % were examined. The compressive and flexural strength increased with the increase in epoxy and filler content; however, above epoxy 20 wt. %, slight change was seen in strength due to increase in epoxy and filler content. Modulus of elasticity also linearly increased with the increase in filler content, but the use of epoxy content beyond 20 wt. % decreased the modulus of elasticity of the mortar. For epoxy content at 10 wt. %, poor bond strength lower than 0.8 MPa was observed, and adding filler at 20 wt. % adversely affected the bond strength, in contrast to the mortars containing epoxy at 15, 20, 25 wt. %. The results indicate that the sand washing waste can be used as potential filler for epoxy resin mortar to obtain better mechanical properties by adding the optimum level of sand washing waste filler.

## 1. Introduction

Concrete structures were assumed to be durable and maintenance free; however, in the past few decades, sign of degradation are becoming apparent [1,2] for various reasons, such as environmental loads, faulty material, construction, and design errors which have caused the deterioration of the structures [3,4,5]. The current practice of the construction industry is inclined towards the repair and retrofit of these deteriorated structures rather than demolition [1,6]. In the United States alone, a significant amount of money is expended in the repair of concrete structures every year [7].

Various materials from conventional Portland cement to polymer binders have been used to perform the repair of concrete structures. Ordinary Portland cement (OPC) is by far the most widely consumed material for the repair and restoration of concrete structures. Despite their vast consumption, OPC-based composites present different shortcomings, such as longer curing time, weak flexural strength, poor bond strength, high shrinkage, low resistance to aggressive environment, and durability problems. These problems of OPC have instigated the use of polymer-based materials as a repair material, which resulted in the introduction of epoxy resin mortar in the repair industry [8].

Epoxy resin mortar has been used as a repair material for the past couple of decades [1,9,10,11,12]; however, their consumption did not grow as anticipated due to the high cost of the epoxy resin and the different properties of the epoxy binder and the substrate concrete which caused compatibility problems [11,13,14]. The properties of the epoxy resin mortar depend on the constituents of the mortar. Hence, it is necessary to optimize the mix proportion of the epoxy resin mortar for feasible repair scheme and to avoid or reduce a mismatch in the properties of the repair mortar and the substrate concrete [15,16,17,18,19]. Different studies used fillers such as fly ash to reduce the epoxy resin dosage and make it more economical [20,21,22,23].

The recycling of industrial wastes such as sand washing waste is important for sustainable development in the construction industry. Sand washing waste is a waste product of the sand production process, and creates a landfill problem. The potential reuse of sand washing waste as a supplementary material for sand is beneficial for reducing the depletion of natural sand [24,25]. Hence, a sand washing waste was utilized in this study as filler for epoxy resin mortar. Experimental study of the effect of the filler on the mechanical properties of epoxy resin mortar was performed.

## 2. Experiment

In this study, a mortar was prepared by using epoxy resin, sand, and filler; the mechanical properties of the mortars with different mix proportions were investigated.

### 2.1. Materials

A two-part epoxy having a Grade 1 viscosity as per ASTM C881 [26] was used as a binder for manufacturing the mortars in this study. Table 1 shows the detailed properties of the epoxy resin and its hardener.

**Figure 1:**
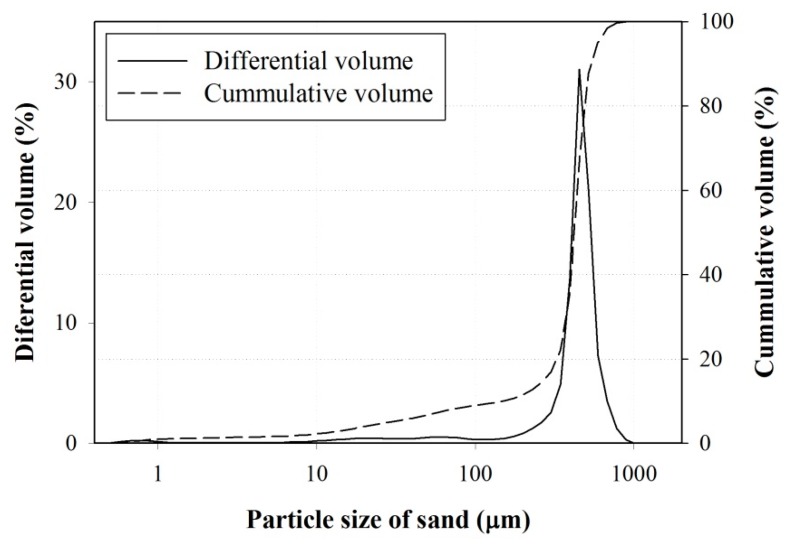
Particle size distribution of sand.

Sand used in this study was purchased from a local store. The physical properties of the sand were characterized according to ASTM C128 [27] and ASTM C29 [28]; the sand was found to have a bulk density of 1.52 g/cm^3^, water absorption of 0.41%, a specific gravity at oven dry condition of 2.6, and a void fraction of 41.28%. Since the epoxy resin mortar was also prepared to repair cracks with small widths, a fine-graded sand with high percentage of particle size less than 600 µm was used. In order to accurately determine the particle size of the sand, a consistent method to that of sand washing waste (laser diffraction technique) was utilized, and Figure 1 presents the particle size distribution.

**Figure 2:**
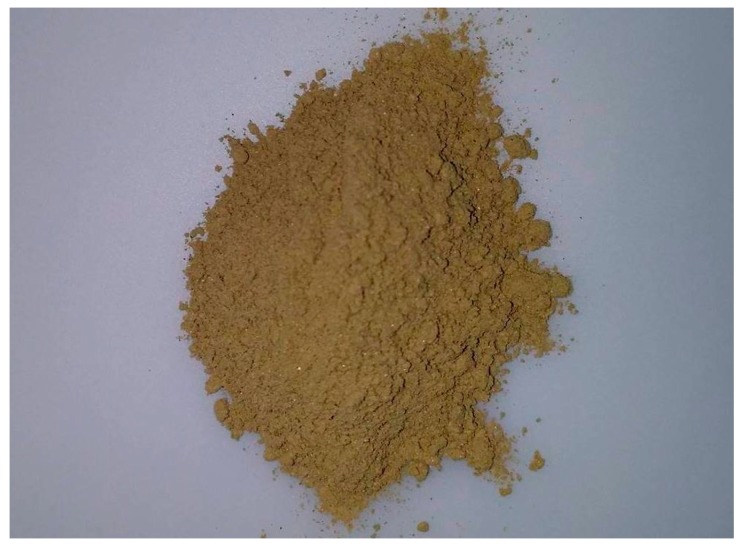
Sand washing waste used as filler for epoxy resin mortar in this study.

**Figure 3:**
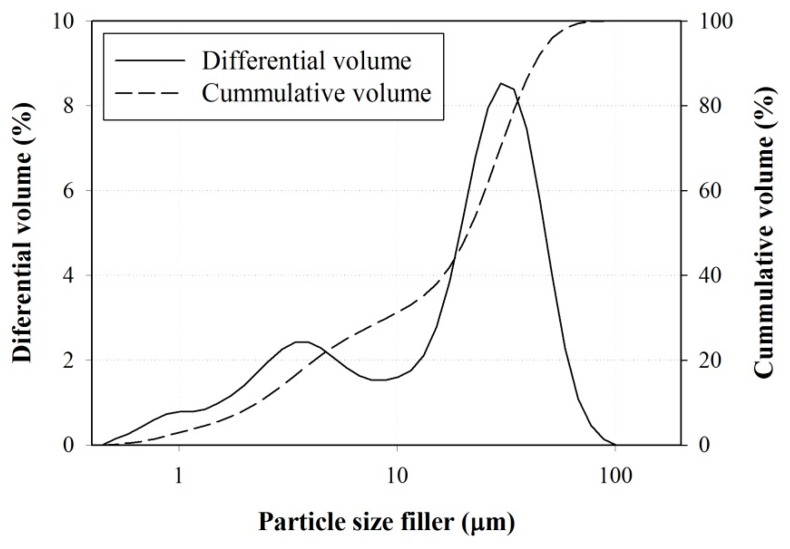
Particle size distribution of filler.

The filler used in this investigation was a sand washing waste that was collected from an aggregates producing plant located in Gyeonggi-do, Korea; the sludge material was a waste product of a sand washing process. The collected sludge was dried at 110 °C to remove any moisture, crushed to break off clusters, and sieved to remove any clusters. The sand washing waste used as filler (Figure 2) had a bulk density of 1.03 g/cm^3^ and specific gravity at oven dry condition of 2.69 according to ASTM C29 and ASTM D854 [29]. The filler particle size distribution was also determined by using LA-950 laser scattering particle size analyzer manufactured by Horiba, Japan, as presented in Figure 3.

**Figure 4:**
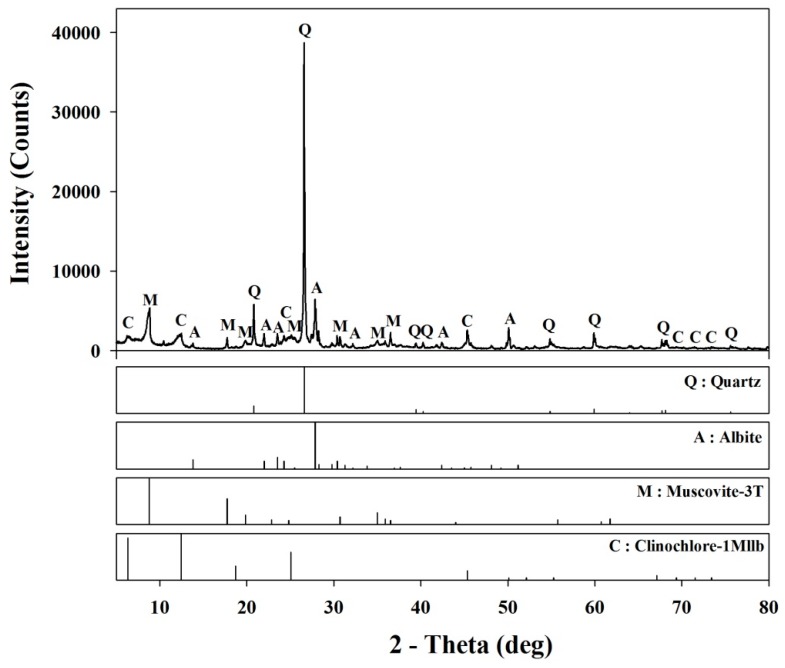
XRD spectra of filler.

Based on the particle size distribution of the sand and the filler, the specific surface area and the mean size were calculated. The specific surface area of sand and filler were found to be 1965.57 m^2^/m^3^ and 140,625.65 m^2^/m^3^, and the mean size was 450.83 μm and 21.12 μm. The chemical composition of the filler and the sand examined using X-ray Fluorescence manufactured by Horiba, Japan is given in Table 2. The filler showed a comparable chemical composition as that of fly ash used as filler to prepare polymer concrete in previous studies [22], and the sand has a majority of silica content. In addition, the X-ray diffraction (XRD) spectra was recorded using a D/MAX 2200 VPC manufactured by Science of Japan Inc. with a continuous scanning speed of 2°/min across a 2θ range of 5°–80° and a Cu/Kα_1_ target (λ = 1.54059 Å), U = 40 kV, and I = 200 mA. The XRD data is shown in Figure 4, which identifies the main crystalline phases in the filler as being quartz, albite, and muscovite.

### 2.2. Mix Proportions

Four mortar groups were prepared with epoxy content at 10%, 15%, 20%, and 25% by weight fraction of the mortar. The minimum epoxy content of 10 wt. % was selected from workability condition, and the maximum content was limited to 25 wt. % due to segregation of the epoxy from the mortar mixture. Filler was added to the mortar mix at 10 and 20 wt. % as sand replacement. The detailed mix proportion of the samples investigated in this study is presented in Table 3.

### 2.3. Test Method

Specimens were prepared to study the effect of filler on the compressive strength, flexural strength and modulus of elasticity, and bond strength of epoxy resin mortar. The sample preparation was performed as follows. Except for control specimens, first dry mixing of sand and filler was performed for 2 min using an automatic mortar mixer to ensure a uniform distribution of the sand and filler. Epoxy resin and its hardener were manually mixed for 1 min in a separate bowl and were added to the mortar mixer. Mixing was continued for another 2 min, and the mortar mixture was casted as per KS F 4043 [30]. Before de-molding was performed after 24 h, the molded specimens were stored in a controlled environmental chamber at 23 °C and 50% RH, and continued curing of the specimen in the same condition was performed for 6 more days.

**Figure 5:**
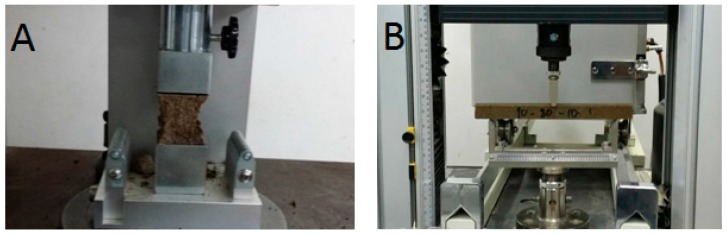
(**A**) Compressive strength and (**B**) Flexural strength and modulus of elasticity through three-point bending tests.

The compressive test of the epoxy resin mortar was conducted according to KS F 4043/EN 12190 [31]. Three compressive strength samples with the dimensions 40 mm × 40 mm × 160 mm were prepared. First, the sample that was cured for seven days was broken into halves through flexure and then the compressive strength test was performed on the broken halves of the samples using a universal testing machine (UTM) at a loading rate of 800 N/min (Figure 5A).

**Figure 6:**
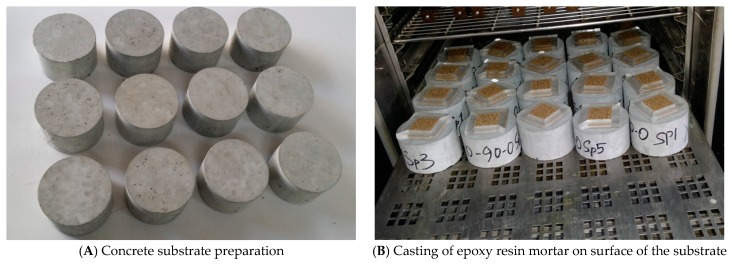

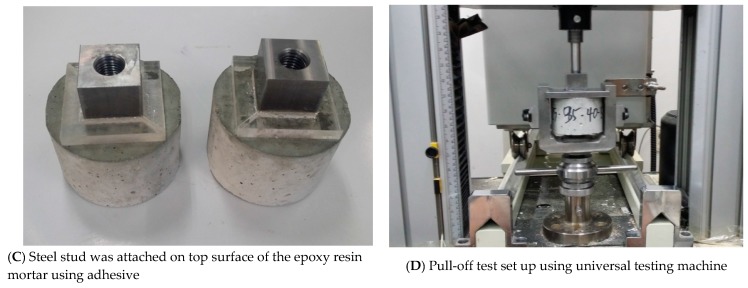
Pull-off test method.

The flexural strength and modulus of elasticity of three epoxy resin mortars of each mix proportion were evaluated in accordance to ASTM C580 [32]. Continuous measurements of the load applied and the corresponding deflection that occurred at the mid span were recorded. The maximum load was used to determine the flexural strength, and the tangent modulus was determined from the load versus deflection curve. KS F 4043 pull-off test method was adopted to measure the bond strength of the epoxy resin mortar to ordinary Portland cement concrete. Three specimens of each mix proportion tested consisted of a concrete of dimensions 100 mm diameter and 70 mm thickness as substrate, with 28-day compressive strength of 50 MPa (Figure 6A). The procedure shown in Figure 5 was used to prepare the specimens and perform the test.

## 3. Results and Discussion

### 3.1. Compressive Strength

**Figure 7:**
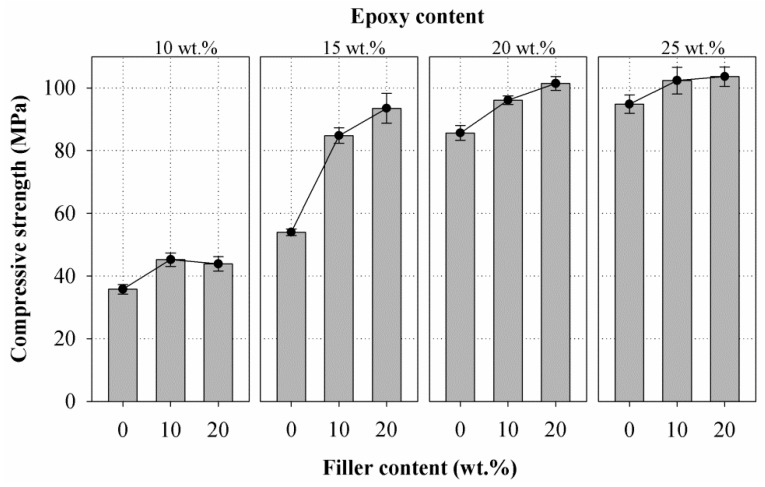
Relationship of compressive strength vs. filler content at different amount of epoxy (10 wt. %, 15 wt. %, 20 wt. %, and 25 wt. %).

Figure 7 presents the compressive strength versus filler content results obtained from mortars prepared with three different filler weight fractions at four various epoxy contents. The results illustrate that the compressive strength of the mortar was affected by both its epoxy and filler content. For mortars prepared with epoxy at 10 wt. %, the compressive strength increased up to 10 wt. % of filler content then leveled off between 10 wt. % and 20 wt. %, while for mortars prepared with epoxy at 15, 20, and 25 wt. %, the compressive strength increased up to 20 wt. % but with a decreasing rate of increase between filler content of 10 wt. % and 20 wt. %. The mortars showed an average improvement in compressive strength of 24.46% for epoxy at 10 wt. %, 65.15% for epoxy at 15 wt. %, 15.28% for epoxy at 20 wt. %, and 8.60% for epoxy at 25 wt. % due to incorporation of filler when compared to the control group. The increase in epoxy content also enhanced the compressive strength of the mortar composite, but the rate of increase diminished at epoxy content of 20 wt. % and 25 wt. %. These can be attributed to the decrease in the void content of the sand with the incorporation of the filler in addition to the epoxy binder [22]. Furthermore, it can be noticed that except for the control group, the increase of epoxy content beyond 15 wt. % appears to enhance the compressive strength of the mortar insignificantly.

The compressive strength is a traditionally examined property of repair material for concrete structures. Mostly it is expected that the ratio of the compressive strength of repair mortar to that of the substrate concrete should be equal to or higher than one. Furthermore, KS F 4043 suggests a minimum compressive strength of 40 MPa for epoxy resin mortars used for repair of concrete structures. All the samples except E10-F0 showed a compressive strength above 40 MPa.

### 3.2. Flexural Strength

**Figure 8:**
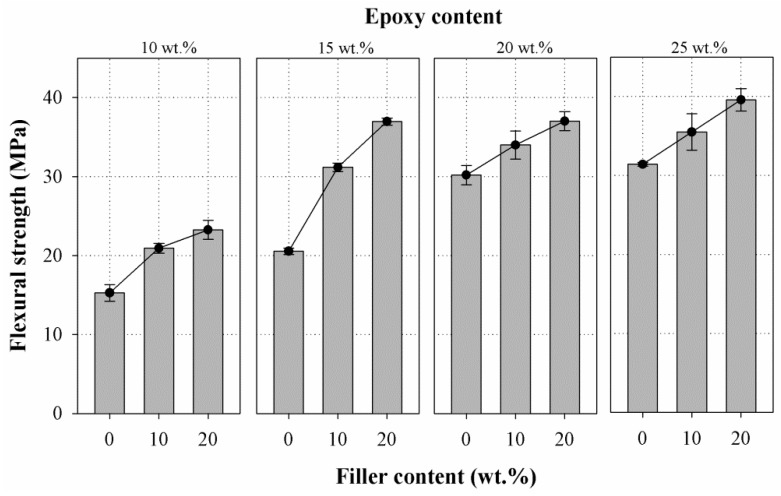
Relationship of flexural strength vs. filler content at different amounts of epoxy (10, 15, 20, and 25 wt. %).

The flexural strength of mortar mixes prepared with varying filler content at given amount of epoxy is presented in Figure 8. The flexural strength of the mortar altered with respect to the filler and epoxy content. Even though the rate of increase slightly decreased between 10 wt. % and 20 wt. %, a more linear increase in flexural strength with R2 value of 0.942 and 0.957 was observed for mortar containing epoxy at 10 wt. % and 15 wt. %. For mortars prepared with epoxy at 20 wt. % and 25 wt. %, the flexural strength increased linearly up to 20 wt. % of filler content with the R2 values of 0.995 and 0.999, respectively. The mortars showed an average rise in flexural strength of 44.6% for epoxy at 10 wt. %, 65.78% for epoxy at 15 wt. %, 17.61% for epoxy at 20 wt. %, and 19.51% for epoxy at 25 wt. % due to incorporation of filler when compared to the control group. The flexural strength of the mortar seems to be slightly beyond 15 wt. % of epoxy content. In general, it can be concluded that a linearly proportional relationship was exhibited between flexural strength and the filler content up to 20 wt. % of the epoxy resin mortar.

The strength of repair materials to withstand a bending load is stated as flexural strength. Generally, the ratio of the flexural strength of the repair mortar and the substrate concrete should be higher than one, whereas KS F 4043 specifies 10 MPa as the minimum flexural strength value for epoxy resin mortars used for restoration of concrete structures. In our study, all mortar mixes prepared exhibited a flexural strength much higher than 10 MPa.

### 3.3. Modulus of Elasticity (MoE)

**Figure 9:**
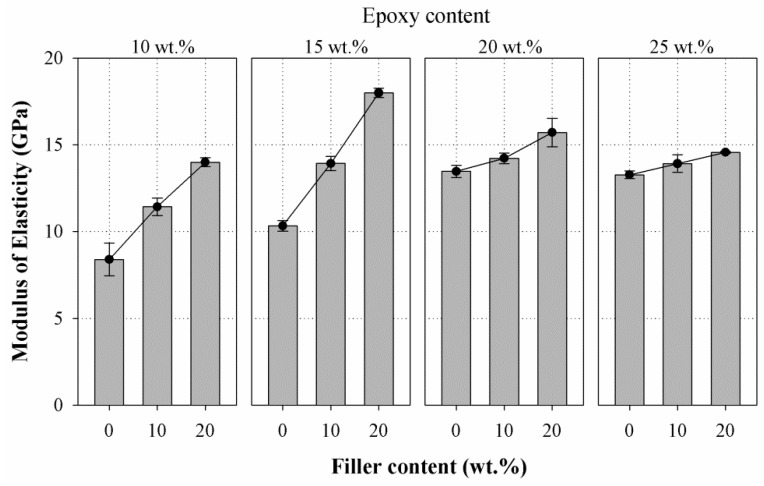
Effect of filler content on the modulus of elasticity of epoxy resin mortars at different amount of epoxy (10, 15, 20, and 25 wt. %).

The tangent modulus of elasticity of the epoxy resin mortars was determined from the load versus deflection relation obtained via three-point bending test. Figure 9 shows the influence of filler content on the MoE of the mortars prepared with different epoxy content (10 wt. %, 15 wt. %, 20 wt. %, and 25 wt. %). It can be noticed that a linear trend was observed as that of flexural strength results between MoE and filler content with R^2^ value of 0.998, 0.999, 0.966, and 0.999 for epoxy content at 10, 15, 20, and 25 wt. %, respectively. The addition of filler resulted in an average increase in MoE of 51.5% for epoxy at 10 wt. %, 54.47% for epoxy at 15 wt. %, 11.10% for epoxy at 20 wt. %, and 7.29% for epoxy at 25 wt. % when compared to the control group. Besides the substantial improvement in the MoE of mortar containing epoxy at 10 wt. % and 15 wt. %, a higher rate of increase in MoE with the increase in filler content was observed when compared to mortars containing epoxy at 20 wt. % and 25 wt. %. The increase in MoE can be attributed to the increased stiffness of the mortar when the filler is added.

**Figure 10:**
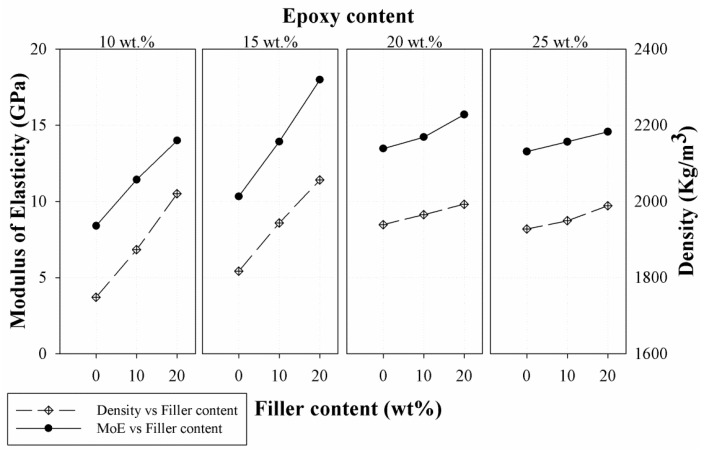
Relationship between modulus of elasticity (MoE) and density of sample.

The density of the epoxy resin mortar three-point bending test beams were determined by measuring the mass and volume in order to understand the relation between the MoE and the density of the hardened beam samples. Figure 10 indicates a plot of the density and MoE versus filler content for the mortars with different epoxy content. The density of the samples showed a similar trend with the increase in filler content as that of the MoE. The results show that a linear correlation with R^2^ value of 0.919 exists between the MoE and the density of the samples.

### 3.4. Bond Strength

The bond strength of epoxy resin mortar to substrate concrete was determined using a pull-off test. A high strength concrete with a 28-days compressive strength of 50 MPa was prepared as a substrate to execute this test. Depending on which section is the weakest, one of the three modes of failures (de-bonding) (failure in the epoxy resin mortar, failure at the interface of epoxy resin mortar and substrate concrete, and failure in the concrete substrate) could occur when the pull-off test on the epoxy resin mortar-bonded concrete is performed.

**Figure 11:**
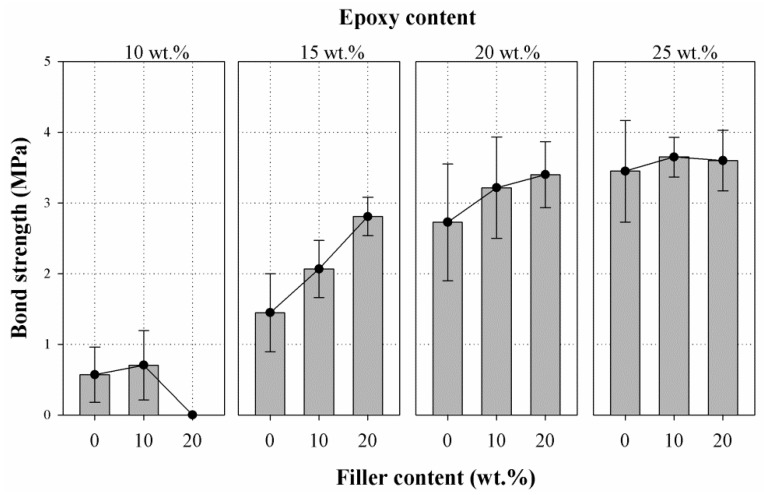
Effect of filler content on the bond strength of epoxy resin mortar at different amounts of epoxy (10, 15, 20, and 25 wt. %) to substrate concrete.

Figure 11 indicates the pull-off test results of concrete bonded with epoxy resin mortar containing varying weight fractions of filler at four different epoxy amounts. For epoxy at 10 wt. %, all the failures occurred at the mortar/substrate concrete interface, and the bond strength seemed to slightly increase with the addition of 10 wt. %; however, the bond strength considerably dropped at 20 wt. % of filler content to the extent that failure of the bond between the epoxy resin mortar and concrete occurred by an insignificant load application. Hence, the bond strength of E10-F20 was specified as zero. The mortars prepared with epoxy at 15 wt. % showed two modes of failure: all the samples except those containing filler at 20 wt. % exhibited a failure in the interface of epoxy resin mortar and substrate concrete, whereas the samples prepared with filler at 20 wt. % demonstrated a failure in the substrate concrete. The bond strength of the mortar at 15 wt. % showed a linear increase with filler content up to 20 wt. %. For epoxy at 20 and 25 wt. %, all the failure occurred in the substrate concrete; the failures seems to occur in the interfacial transition zone of the coarse aggregate in the substrate concrete. Hence, the effect of filler content on the adhesion property of epoxy resin mortar to concrete was not clearly understood, because the failure was mainly governed by the intrinsic property of the concrete [33,34].

Bond strength of repair material is an important property that holds the repair mortar and the substrate concrete bonded as a unit. Hence, in order to have a durable repair work of concrete structures, the mortars used should have adequate adhesion to avoid bond failure between the repair and the substrate because of the stresses developed due to internal and external loads. KS F 4043 suggests a minimum of 1.5 MPa bond strength of the epoxy resin mortar used for the maintenance of concrete structure. In this study, the epoxy resin mortar prepared with 10 wt. % epoxy showed poor bond strength lower than 0.8 MPa. Whereas all the samples with epoxy at 15, 20, and 25 wt. % showed a bond strength higher than 1.5 MPa.

## 4. Conclusions

This study investigated the effect of filler on the mechanical and adhesion properties of epoxy resin mortar. The following conclusions were drawn based on the experimental results obtained.

(1) The compressive strength, flexural strength and modulus of elasticity value of epoxy mortar containing filler up to 20 wt. % improved by an average of 1.08–1.66, 1.18–1.66, and 1.07–1.54 times, respectively, at different weight fraction of epoxy in the range of 10–25 wt. % when compared to the control specimens.
(2) It was observed that when filler is used at optimum level, it can improve bond strength; all the mortars except those prepared with epoxy at 10 wt. % showed a good bond strength higher than 1.5 MPa.
(3) It can be established that sand washing waste can be used as potential filler for epoxy resin mortar to obtain better compressive, flexural, stiffness, and bond strength.
(4) This study does not cover all the parameters required to examine repair materials for concrete structures; further investigations on compatibility issues such as dimensional change stability of the epoxy resin mortar containing sand washing waste filler is required.

## Figures and Tables

**Table 1:** Properties of epoxy resin and its hardener.

Type	Epoxy Resin	Hardener
Mixing proportion	3	1
Specific gravity	1.14 ± 0.1	1.02 ± 0.1
Color	Colorless	Brown
Viscosity (mPa·s)	550 ± 50	
Pot life (min)	30 ± 10 at 23 °C	
Hardening time (h)	24–36 at 23 °C	

**Table 2:** Chemical composition of sand and filler (wt. %).

Chemical Composition	SiO_2_	Al_2_O_3_	K_2_O	Fe_2_O_3_	TiO_2_	MnO_2_	CaO
Sand	87.720	5.915	6.277	-	-	-	-
Filler	70.993	18.070	1.792	6.450	0.805	0.282	1.607

**Table 3:** Mix proportion by weight percent (wt. %) and specimen notation.

Specimen *	Epoxy (E)	Sand (S)	Sand Washing Waste Filler (F)
E10-F0	10.00	90.00	0.00
E10-F10	10.00	80.00	10.00
E10-F20	10.00	70.00	20.00
E15-F0	15.00	85.00	0.00
E15-F10	15.00	75.00	10.00
E15-F20	15.00	65.00	20.00
E20-F0	20.00	80.00	0.00
E20-F10	20.00	70.00	10.00
E20-F20	20.00	60.00	20.00
E25-F0	25.00	75.00	0.00
E25-F10	25.00	65.00	10.00
E25-F20	25.00	55.00	20.00

* Specimen notation includes two parts. The first part is used to identify the epoxy/mortar ratio by weight, and the other is used to identify the filler/mortar ratio by weight. For example, E20-F10 is a specimen having epoxy/mortar = 20% and filler/(sand + filler) = 10%.
